# A link between migraine and prolactin: the way forward

**DOI:** 10.2144/fsoa-2021-0047

**Published:** 2021-09-22

**Authors:** Parisa Gazerani

**Affiliations:** 1 Department of Life Sciences & Health, Faculty of Health Sciences, Oslo Metropolitan University, 0130 Oslo, Norway; 2 Department of Health Science & Technology, Faculty of Medicine, Aalborg University, 9220 Aalborg E, Denmark

**Keywords:** female, headache, hormone, male, migraine, prolactin, prolactin receptor, sex

## Abstract

Migraine is an incapacitating neurological disorder that predominantly affects women. Sex and other hormones (e.g., oxytocin, and prolactin) may play a role in sexual dimorphic features of migraine. Initially, prolactin was recognized for its modulatory action in milk production and secretion; later, its roles in the regulation of the endocrine, immune and nervous systems were discovered. Higher prolactin levels in individuals with migraine were found in earlier studies, with a female sex-dominant trend. Studies that are more recent have identified that the expression of prolactin receptor in response to neuronal excitability and stress depends on sex with a dominant role in females. These findings have opened up potentials for explanation of sex-related pathophysiology of migraine, but have left some unanswered questions. This focused review examines the past and present of the link between prolactin and migraine, and presents open questions and directions for future experimental and clinical efforts.

## Lay abstract

Sex hormones (e.g., estrogen and progesterone) have been investigated to explain the sex-related manifestation of migraine, which is predominant in females. Prolactin is known for promoting lactation, but accumulating evidence supports that it can promote pain in females. An increasing number of studies have shown that the expression of a prolactin receptor in female nociceptors and their responses to external stimuli such as stress are different, which can help explain the female sex-dominant feature of migraine. In this focused review, the current knowledge is presented and the directions where prolactin research in migraine may evolve are proposed. The ultimate goal is to shape an overview toward considering sex-based treatments for migraine with highlighting the role of prolactin.

Migraine is a common incapacitating disorder that often presents with autonomic and neurologic symptoms of severe headaches that occur recurrently [1]. Women between the ages of 30 and 45 years, in particular, are affected, with as many as 25–30% of the general female population affected [2] compared with 8% of men affected, and therefore, the burden of this disorder is higher in women [3]. There are several interpretations for the sexual dimorphic etiology of migraine [4], and hence, proposals for sex-based treatments of migraine have emerged [5]. Observations of changes in women with migraine during menstrual cycles, pregnancy, and menopause have suggested that a hormonal component contributes to the sexual dimorphic pathophysiology of migraine [6]. Although most studies have been conducted on the role of sex hormones (estrogen, progesterone and testosterone) [7], several other hormones have also been investigated, including growth hormone, somatostatin, oxytocin and prolactin [8]. Historically, two lines of evidence have been presented that link prolactin and migraine. First, in hyperprolactinemia, headaches with migraine characteristics have been described and normalization of the prolactin level has alleviated headaches [9,10]. Moreover, in migraine patients, higher levels of prolactin have been identified that were also responsive to drugs lowering the prolactin level [11,12]. Second, a new line of evidence has shown that prolactin receptor expression and neuronal responsiveness to external stimuli and stress are different in female and male laboratory animals, which can explain some mechanisms underlying sex-related differences in migraine, with a spotlight on prolactin [8,13–16]. These findings have opened up potentials for explanation of sex-related pathophysiology of migraine, with a focus on prolactin, but have left some unanswered questions. This focused review examines the past and present of the link between prolactin and migraine, and presents current open questions and potential directions for future experimental and clinical efforts.

In the following, after a short overview to prolactin, key findings from clinical and basic studies about a link between prolactin and pain/headache/migraine are presented and subsequently a number of open questions are raised to stimulate and guide future research under two broad headings: serological levels of prolactin in patients with migraine and migraine-like headaches in patients with high circulatory levels of prolactin; basic research of prolactin receptor expression and neuronal responsiveness to external stressors that has unfold some mechanisms underlying sexual dimorphic etiology of prolactin-pain association. These two categories by no means exhaust all potential directions of future studies, but the selection is to represent main gaps in the current knowledge and most immediate next directions.

## Prolactin

Human prolactin is a polypeptide that is composed of 199 amino acids and produced by the lactotroph cells of the anterior pituitary gland [17]. Prolactin is also produced by many other cell types (e.g., mammary glands, ovaries, prostate gland, testes, endothelial cells and adipose tissue) [18]. Although prolactin was first described in the 1930s for its role in controlling milk production and secretion, currently, it is considered a multifunctional endocrine hormone with more than 300 physiological functions [19]. Prolactin plays regulatory and modulatory roles in the endocrine, immune and nervous systems and has been associated with the etiology of pain and headaches [13,20]. Prolactin secretion is influenced by many factors, including physiologic factors (e.g., pregnancy, sleep and stress), pathologic factors (e.g., pituitary adenoma, hypothyroidism and renal failure), and some drugs (e.g., antipsychotics, antidepressants, prokinetics, morphine and antihypertensive agents). Prolactin levels follow a circadian rhythm [21], adjusted by the hypothalamus, which is also believed to be involved in the onset of migraines [22]. Premonitory symptoms (e.g., food craving and mood swing) have been linked to hypothalamic dysfunction [23], and perturbations in the hypothalamic–pituitary–gonadal (HPG) axis have been associated with menstrual migraine [24,25].

Prolactin performs its biological function by acting on the prolactin receptor [26]. The prolactin receptor is a type-I cytokine receptor that mainly activates the Janus tyrosine kinase 2 (JAK2)/signal transducers and activators of transcription 5 (STAT5) signaling pathway; however, other signal cascades can also be initiated [27,28]. The prolactin receptor comprises multiple isoforms, including long, intermediate and short isoforms that are produced by the human prolactin receptor gene, located on chromosome 5 [29]. The prolactin receptor in humans is distinct from rodents, which is an essential aspect to consider when translating data from the preclinical stage to humans. In humans, the prolactin receptor is activated by prolactin and other hormones, for example, growth hormone and placental lactogen (PL) [30]. The latter is secreted in animals and humans during pregnancy [20]. Therefore, to determine the mechanisms underlying diseases and drug development, this cross-reactivity in humans must be considered. Interestingly, pituitary gland hormones, including adrenocorticotropic hormone (ACTH), growth hormone (GH), luteinizing hormone (LH), follicle-stimulating hormone and prolactin have all been found to play critical roles in pain. The regulatory role of prolactin has been found in nervous system processes such as stress responses, anxiety, neurogenesis, orofacial pain and migraine [4].

## Prolactin & migraine: clinical research

### Prolactin levels in patients with migraine

One line of evidence supporting the view that prolactin plays a crucial role in the pathophysiology of migraine is an investigation of prolactin circulatory levels in migraine [31]. A systematic review followed by a meta-analysis [11] summarized the available literature since the 1970s on prolactin blood levels in patients with migraine. In this systematic review, 13 studies were found to be relevant, with a total number of 460 migraineurs and 429 healthy controls, with a high heterogeneity among the studies. For the meta-analysis, a random effects model (i.e., variance components model) was preformed and the findings demonstrated that the blood levels of prolactin were higher in patients with migraine compared with healthy controls [11]. However, some studies have shown that prolactin level decreased during a migraine attack; for example, a study comparing 20 men with migraine with 20 men without migraine reported that prolactin levels were lower in patients with migraine [32]. According to Masoud *et al.* [31], such controversial results may have originated from small size studies. Other studies have reported that migraine patients who did not have pituitary adenomas did not show any higher serum prolactin levels compared with healthy controls [33] and have concluded that, potentially, only under pathological conditions such as pituitary adenomas, hyperprolactinemia was present and thereafter excess prolactin could trigger a migraine.

To identify whether type of migraine can influence the outcome, one study [34] compared serum levels of prolactin in female subjects with episodic migraine versus chronic migraine. This study was performed in 2011–2012 and included 114 females with migraines. The authors identified that prolactin serum levels and the presence of hyperprolactinemia were higher in females with chronic migraine than those with episodic migraine. In another study, however, lower prolactin serum levels were found during the acute phase of migraine [31]. Cavestro *et al.* [12] reported that, among 27 chronic migraine patients (with a history of episodic migraine), seven patients were found with increased prolactin levels, and therefore, they considered that prolactin might be a potential worsening factor for migraine. This was based on the findings that the severity and frequency of headaches had changed. Microprolactinoma was observed in only one of the patients. Patients with chronic migraine, in this study, responded to a D_2_ dopamine receptor agonist, in other words, cabergoline, which was used to block prolactin secretion [12]. The rationale behind the use of this agent was that prolactin secretion was known to be controlled by dopamine [31]. Dopamine agonists including bromocriptine and carbidopa/levodopa have been used to successfully treat migraine resulting from high prolactin levels [35,36].

Peres *et al.* [37], in a study of patients with chronic migraine (n = 17), collected blood samples every hour for 12 h to investigate the role of the hypothalamus in chronic migraine, by measuring melatonin, prolactin, growth hormone and cortisol levels. In [37], Peres *et al.* found that nocturnal prolactin peaks were lower among patients with chronic migraine.

Collectively, the studies presented above support the existence of a link between migraine and prolactin, however, it remains to be exactly elucidated how elevated endogenous prolactin levels can cause migraine and why different responses are seen in episodic versus chronic migraine. In addition, number of questions remained unanswered related to alterations of the other features of the somatosensory system [38,39], for example, sensitivity to touch, light and temperature. It is not yet clear as if high level of prolactin can only influence the headache phase of a migraine attack or can play a role in prodrome symptoms and aura phases, for example, in migraine with aura.

When it comes to hormones and pain, transgender and transsexual individuals must not be neglected. These individuals undergo the transition period, which includes hormone therapy, surgery or both [40]. Set aside the prolactin and other hormones, very limited information is available related to sex hormone therapy and alterations in pain perception. One study [41] presented that cross-sex hormone administration altered pain perception in transsexual individuals; 29.8% of MtF reported painful conditions, interestingly manifested as headaches, breast and musculoskeletal pain, whereas 61.5% of FtM reported pain, which was improved after the administration of testosterone. The percentages show that not all individuals are affected, and the reason for this remains unknown. Data on migraine in transgenders are extremely limited. A questionnaire survey in Holland [42] found a prevalence of 26% migraine in transgender women. The study consisted of individuals completed reassignment surgery receiving anti-androgens and estrogens for gaining their sex characteristics. Research within the transgender domain is gradually expanding, and future studies can reveal more information related to cross talk between different hormones and pain/headache characteristics in these individuals. It is therefore suggested that next studies to broaden the investigation of hormones and to potentially include prolactin while studying migraine/headache/pain in this population.

### Hyperprolactinemia & migraine-like headaches

Hyperprolactinemia often occurs following adenomas [43,44]. However, hyperprolactinemia can also be a result of other diseases, physiological conditions or consumption of some medications [45]. Sleep, pregnancy, stress and breast examination are conditions known to elevate prolactin levels. Some disorders also cause elevated levels of prolactin, for example, hypothyroidism, renal failure and cirrhosis. Several classes of drugs have been identified to increase prolactin levels, for example, tricyclic antidepressants and antipsychotic drugs [45,46]. Interestingly, there is a higher frequency of antipsychotic-induced hyperprolactinemia in women compared with men [47]. The prevalence of hyperprolactinemia is high in women, in other words, 5% in infertile women, 9% in those with galactorrhea and 25% in women with amenorrhea [48]; however, hyperprolactinemia is also present in men and causes hypogonadism in both men and women [49,50]. Men with hyperprolactinemia have a lower sexual desire (libido) and dysfunction in erection [50].

Adenomas of the pituitary gland are classified as nonfunctional (silent) and functional (hormone-secreting) forms with regard to secreted hormones [51,52]. In functional adenomas, in particular, prolactin-secreting tumors [53], facial sensitivity to light touch (allodynia), and headaches commonly occur [8]. Prolactinoma-associated headaches have been reported to occur in 37–83% of cases [53] and this presents that secondary headaches as a consequence of pituitary diseases are common [54]. These headaches have been described as migraine-like [36], cluster headaches [55], paroxysmal hemicrania [56] and short-lasting unilateral neuralgiform headache attacks with conjunctival injection and tearing (SUNCT) [57–60]. Headaches with migraine characteristics or worsening of a known migraine have been reported in females with prolactinoma [59]. Early studies have suggested that pituitary tumors could cause secondary headaches because of the tumor mass and pressure on nerves [54,61]. On the one hand, Levy *et al.* [59,62] reported that, in microadenomas, where the tumor size is very small, patients still experienced severe disabling headaches; while, in macroadenomas, where the tumor size is large, some patients did not experience headaches. This observation suggested that size was not the only driver of head pain and that tumor mass was not correlated with the presence or intensity of the headache [59,62]. Others [9] who also described the occurrence of headaches in patients with microprolactinoma have supported this notion that prolactin might exert a modulatory effect on neuronal excitability rather than tumor-related factors such as size [13].

Usually, prolactinoma-associated headaches are localized on the same side as the tumor and respond well to dopamine agonists [9,53]. A longitudinal study [10] evaluated patients with hyperprolactinemia for presenting the characteristic of headache before and after treatment by D_2_ agonists. In this study, prolactin levels were decreased after treatment and the pain subsided. Other studies have also reported a reduction in pain by the application of dopamine agonists [9,12,53,63]. Some cases of migraines have been treated with bromocriptine, a D_2_ agonist that inhibits prolactin release [35,36]. On the other hand, there are two cases where the administration of dopamine agonist led to headache increase [64]. Therefore, controversy still exists and the reason is not clear.

Moderation of hyperprolactinemia is not limited to the application of D_2_ agonists. A recent study [65] has proposed that vitamin B_6_ could reduce prolactin levels, and therefore, the effectiveness of vitamin B_6_ for reducing serum prolactin in patients with hyperprolactinemia compared with cabergoline was evaluated. The results showed that vitamin B_6_ was comparable to cabergoline in reducing prolactin levels; however, implication of these findings for patients with migraine and other headaches related to hyperprolactinemia is not clear and hence needs further investigation.

Collectively, the studies presented above support the existence of a link between migraine-like headaches and hyperprolactinemia, however, it remains to be exactly elucidated if this knowledge can be used at clinic for diagnosis of a secondary migraine-like headache form a primary migraine headache and how. It is also worthwhile to mention that considering multifunctional role of prolactin and comorbid conditions in migraine, how being at a certain age or under a single or multiple simultaneous conditions can change bodily responses. It remains to be determined how several factors interacting with each other to create one specific response and how this response can guide for treatment strategies to be specific and safe. Most studies focus on exploring role of one factor at a time, and this limits the understanding of complex relationship that exists between one and other hormones, influential role of age-related factors, and critical role of comorbid conditions in migraine [2]. It is immature to consider that sexual dimorphism of migraine can be explained by one factor. Therefore, next studies must employ novel and innovative designs that can employ multifactorial nature of interacting factors while studying migraine.

## Prolactin & migraine: basic research

### Association between prolactin & pain: potential underlying mechanisms

Prolactin receptor is expressed in sensory neurons of sensory ganglia (e.g., trigeminal ganglia) in both rats and mice [38]. Expression of prolactin receptors has also been reported in dural afferent nerve fibers. These structures are known to contribute in nociception and pathogenesis of migraine headaches. Current data show that prolactin short isoforms enhance nociceptor excitability and pain while long isoforms protect against it, but this only occurs in female laboratory animals. Accumulating evidence [66] supports that prolactin and the prolactin receptor system function under a female-selective mechanism. Here, the key findings are presented.

Chen *et al.* [39] demonstrated the role of prolactin receptor in the regulation of nociceptor sensitization to be a female-selective role. They found higher levels of prolactin and a higher expression of prolactin receptor in female animals. They also detected that the long isoform was the protective factor responsible for sensitization of nociceptors. They observed that cabergoline reduced the prolactin level, which led to upregulation of the long isoform of prolactin receptor in females. Besides pharmacological modulation, gene therapies that target the long isoform have also been effective against pain in a female-selective manner [39]. Interestingly, the expression of prolactin receptors in dural afferent nerve fibers has only been found it in female mice [8]. In addition, application of prolactin has increased the excitability of dural afferent nerve fibers only in females [8]. Subsequently, studies have shown that the application of prolactin could potentiate the transient receptor potential cation channel (TRP) subfamily V member 1 (TRPV1) in trigeminal ganglion neurons in rats [38] and subfamilies V1, A1 and M8 in dorsal root ganglion neurons in mice [67,68]. This presents the importance of considering animal species and that the receptor sub types might be differentially activated. In search for downstream pathways of TRP channels activation, studies have revealed that the sensitization of TRPV1 by prolactin activates the short isoform of the prolactin receptor and activates the downstream pathway of protein kinase Cδ (PK Cδ) and phosphoinositide 3-kinase (PI3K) [67,68]. Interestingly, TRPV1 can be sensitized with a 40-fold lower concentration of prolactin in females [67,68].

A key molecule in nociception and in particular migraine pathogenesis is calcitonin gene-related peptide (CGRP) [69]. This has led to drug discovery for migraine and CGRP-based novel drugs have emerged for the management of migraine [70,71]. To identify how prolactin can interact with this key molecule, cultures of trigeminal ganglion neurons were pretreated and tested with prolactin to find if capsaicin-evoked release of CGRP in these cultures are changed [38]. The results showed that prolactin enhanced CGRP release in this *in vitro* test system. This observation proposed that higher levels of prolactin may sensitize sensory neurons, potentially through CGRP, leading to a sexual dimorphic susceptibility to migraine. However, no data are available to confirm similar findings in humans, and the existence of a sex-based response associated with novel drugs (monoclonal antibodies against CGRP or its receptor) or CGRP inhibitors (drug class of gepants). This can be investigated when accumulating data are becoming available following administration of those new drugs. Considering pharmacokinetic parameters and differences between females and males, both in laboratory animals and humans, future basic and clinical studies may unfold some characteristics of sex-related responses [5,72].

Besides *in vitro* studies, a few *in vivo* studies have also evaluated prolactin and its receptor isoforms in migraine models [73] and collectively the outcomes support the proposed potential for migraine development as a predominantly female disorder. For example, when prolactin was injected into the dura of female animals [74], allodynia was evident in the periorbital area (lower facial withdrawal thresholds in response to von Frey filaments). In these animals, higher grimace scores were also recorded. In female mice, already sensitized, for example, by opioids [39] or the inflammatory agent IL-6 [16], even lower doses of prolactin could elicit a profound pain response. In search for identification of interaction between sex hormones and prolactin, behavioral tests have also shown that administration of prolactin can induce acute nociception in intact females but not in ovariectomized rats [38]. This finding highlights the importance of estrogen in the activity of prolactin in rodents, but we still do not know if similar phenomenon occurs in humans.

Stress is a risk factor for migraine attack, and a known factor leading to the release of prolactin [75]. Basic researchers attempted to identify how stress can affect prolactin release in a behavioral test of stress. Transient restrain causes stress in animals, and has been used as a model of stress in laboratory animals. Researchers used this model and found that both female and male animals released prolactin [76]; however, in females, a slightly higher release was seen [77]. Interestingly, hypersensitivity levels of stressed animals that received bromocriptine returned to baseline faster. Studies have also found that knockout of the prolactin receptor from Nav1.8-expressing nociceptors did not affect males but reduced the priming response to nitric oxide donors after stress in knockout females [8,16,78]. Recent data in mice have indicated that different stressors, such as surgery and inflammation, could elevate prolactin levels [77,79] predominantly in females. It has been suggested that prolactin release in these cases most likely originated from immune cells [80]. Collectively, these data demonstrate that stress-induced prolactin release and hypersensitivity responses are predominant in females, regardless of type of stressors.

While the findings presented above are fascinating, there are number of unanswered questions for the next steps in the basic research. Available data show that prolactin acts indirectly to sensitize sensory neurons, for example, via TRP channels, CGRP or other unknown paths. However, prolactin can activate prolactin receptor, which is also expressed in nonneuronal cells and that can also promote neuronal sensitization via crosstalk between neuronal and non-neuronal cells. Cell specificity of responses to prolactin has just started to be investigated but can present how this feature can influence differentiated responsiveness and what can that be used for, in terms of modulation or therapeutic purposes.

The focus of this review is migraine, but it is interesting to identify how different are orofacial pain conditions [8] compared with other types of pains, for example low back pain, while the prolactin is being investigated. This idea stem from studies [38,68,81] that have reported differentiation in dorsal root ganglion versus trigeminal ganglion in terms of mRNA expression of prolactin in male and female animals.

Two other puzzling conditions are pregnancy and lactation, where prolactin is high, but no amplification of pain has been recorded. Proposed mechanisms are T-cell-mediated endogenous opioid analgesia occurring in the third trimester [82] and higher oxytocin levels as a consequence of breastfeeding during the nursing period [83]. The theory is that prolonged high concentrations of prolactin might reduce inflammation and pain [84,85]; however, these theories need further investigation.

Last, but not least, one needs to consider that prolactinomas add complexity because under this condition, headaches develop regardless of gender in humans [53] and very high levels of prolactin can also sensitize sensory neurons in male mice [68]. Therefore, it is highly important to investigate further and understand how prolactin did not show a role in pain sensitivity in male animals in the above-mentioned studies. It is not clear whether this can be related to pain conditions, amount of prolactin, or specific female-dominant mediating pathways for example specific contribution of CGRP [86,87].

## Conclusion & future perspective

In view of the current literature of basic research and clinical studies, prolactin does seem to have a role in migraine and its role carries some sexual dimorphic features. However, there are several unanswered questions to be addressed in the next steps. Table 1 summarizes the points discussed above to present a structured overview for the path forward in studying prolactin and migraine in future basic and clinical investigations.

**Table 1.** Proposed points of considerations for future studies of association between prolactin and migraine headache (or pain).

Basic study	Human study
• Considering translational aspects in design: ○ Prolactin receptor expression in human sensory neurons ○ Prolactin receptor expression in different pain conditions (disease selective?) ○ Higher pain sensitivities to prolactin in female humans (modality selective?) ○ Experimental migraine provocation, or neuro inflammation models ○ Biomarkers? • Considering multi-dimensional type design: ○ Including a number of transmitters and hormones to study interactions among them and with prolactin. ○ Application of several risk factors to study simultaneous interactions, overlapping and interacting pathways • Considering confounding factors: ○ Age ○ Sex ○ Obesity ○ Conditions such as lactation and pregnancy ○ Acute vs chronic ○ Diet ○ Sleep ○ Drugs • Considering targeting ○ Include monitoring of unwanted consequences ○ Include cross interaction of hormones with immune and nervous system • Considering sex-based therapeutic strategies ○ Targeting the prolactin receptor in female and male models (include dose response) ○ Potential for antibodies against prolactin or its receptor? ○ Gene therapy? • Considering internal and external models of hyperprolactinemia ○ Hyperprolactinemia, migraine like behavior and targeting in female and male • Considering cross sex studies mimicking transgender and transsexual	• Considering prolactin as a biomarker (specificity-sensitivity) for prognostic, diagnostic or response to treatment purposes • Considering migraine types (aura, no aura, episodic, chronic) and other headaches (tension type headache, cluster headache) • Considering bio psycho social view in study designs • Considering confounding factors: ○ Age ○ Sex ○ Obesity ○ Conditions such as lactation and pregnancy ○ Diet ○ Sleep ○ Stress ○ Drugs • Considering targeting: ○ Include monitoring of unwanted consequences ○ Include cross interaction of hormones with immune and nervous system • Considering sex-based therapeutic strategies • Considering comorbidities (psychiatric disorders, other pain conditions), other conditions (pregnancy-breast feeding) • Considering hormones and migraine in transgender and transsexual • Considering longitudinal studies, prolactin effect along life span • Considering antimigraine effects on prolactin levels

Most likely, prolactin and other pituitary hormones remain attractive targets for more studies in the fields of neuroscience, immunology, reproductive system and metabolism, all somehow contributing to migraine pathophysiology.

**Executive summary**

- An association between prolactin and migraine has been presented in the literature, and females seem to be affected differently compared with males.
- The current literature, based on a systematic review and meta-analysis, supports the notion that prolactin is elevated in patients with migraines.
- There is evidence that reducing prolactin levels could subside headaches that are associated with higher levels of prolactin.
- Recent basic experimental data present that the expression and activity of prolactin receptor are distinct in females compared with males.
- Animal studies have provided valuable data on the sexual dimorphic understanding of the prolactin and prolactin receptor interactions and downstream signaling pathways.
- Translational basic studies and further studies in humans would reveal whether the presented data are applicable in humans to explain why migraine is more prevalent among females and how the information can advance strategies for management of migraine.
- The efficacy and safety aspects of targeting prolactin and its receptor are open to further investigation, although this path might be challenging due to the multidimensional role of prolactin and its interaction with other transmitters and hormones.
